# Characteristics and key differences between patient populations receiving imaging modalities for coronary artery disease diagnosis in the US

**DOI:** 10.1186/s12872-023-03218-7

**Published:** 2023-05-15

**Authors:** Matthieu Pelletier-Galarneau, Emily Vandenbroucke, Minyi Lu, Olivia Li

**Affiliations:** 1 Department of Medical Imaging, Montreal Heart Institute, Montreal, QC H1T 1C8 Canada; 2 GE Healthcare, Marlborough, MA US; 3 Clarivate Analytics, Toronto, ON Canada

**Keywords:** Diagnostic imaging, Coronary artery disease, Coronary angiography, Myocardial perfusion imaging, Single-photon emission computed tomography

## Abstract

**Background:**

There are limited data on the impact of imaging modality selection for the assessment of coronary artery disease (CAD) risk on downstream resource utilisation. This study sought to identify differences between patient populations in the US undergoing stress echocardiography, single-photon emission computed tomography (SPECT) myocardial perfusion imaging (MPI), positron emission tomography (PET) MPI, and coronary computed tomography angiography (cCTA) for the assessment of CAD risk, and associated physician referral patterns.

**Methods:**

Claims and electronic health records data for 2.5 million US patients who received stress echocardiography, cCTA, SPECT MPI or PET MPI between January 2016 and March 2018, from the Decision Resources Group Real-World Evidence US Data Repository, were analysed. Patients were stratified into suspected and existing CAD cohorts, and further stratified by pre-test risk and presence and recency of interventions or acute cardiac events (within 1–2 years pre-index test). Linear and logistic regression were used to compare numeric and categorical variables.

**Results:**

Physicians were more likely to refer patients to standalone SPECT MPI (77%) and stress echocardiography (18%) than PET MPI (3%) and cCTA (2%). Overall, 43% of physicians referred more than 90% of their patients to standalone SPECT MPI. Just 3%, 1% and 1% of physicians referred more than 90% of their patients to stress echocardiography, PET MPI or cCTA. At the aggregated imaging level, patients who underwent stress echocardiography or cCTA had similar comorbidity profiles. Comorbidity profiles were also similar for patients who underwent SPECT MPI and PET MPI.

**Conclusion:**

Most patients underwent SPECT MPI at the index date, with very few undergoing PET MPI or cCTA. Patients who underwent cCTA at the index date were more likely to undergo additional imaging tests compared with those who underwent other imaging modalities. Further evidence is needed to understand factors influencing imaging test selection across patient populations.

**Supplementary Information:**

The online version contains supplementary material available at 10.1186/s12872-023-03218-7.

## Background

Coronary artery disease (CAD) persists as a major cause of mortality and disability worldwide [1–3]. CAD is the cause of one-third of all deaths in people aged ≥ 35, with a prevalence of 5–8% globally [2, 4]. Low- and middle-income countries bear a greater burden of CAD, which accounts for 7 million deaths and 129 million disability adjusted life years annually in these countries [2]. In the United States (US), CAD affects 16.8 million people, with nearly 8 million cases of myocardial infarction [5].

CAD is also associated with a substantial economic burden; between 2016 and 2017, heart diseases including CAD cost the US $363 billion [6]. In the European Union, CAD accounts for 27% of the total cardiovascular disease burden (€169 billion annually), [7] and in the United Kingdom, the total economic burden of CAD amounts to £7.06 billion annually [8]. A systematic review assessing the economic burden of CAD in low- and middle-income countries reported that the average monthly treatment cost associated with CAD was between $300 and $1,000 [9].

Invasive coronary angiography (ICA) has traditionally been considered the gold standard for diagnosing CAD. However, due to its invasive nature, the technique is associated with risks, including local vascular injuries, conduction disturbances, and contrast induced nephropathy [10, 11]. It has also been suggested that direct referral to ICA leads to higher rates of inappropriate percutaneous coronary intervention (PCI) [12, 13]. In addition, the high cost of performing ICA demands efficient use of this procedure [14, 15].

Recent years have seen a shift towards the use of ICA following assessment of CAD risk using non-invasive imaging modalities, including stress echocardiography, single-photon emission computed tomography (SPECT) myocardial perfusion imaging (MPI), positron emission tomography (PET) MPI, and coronary computed tomography angiography (cCTA) [10, 16–19]. Despite thousands of publications discussing the optimal approach for the diagnosis of CAD, there remains no consensus [20] .Diagnostic imaging guidelines in CAD outline the value of different testing modalities, dependent on patient presentation and clinical profile [16, 19]. However, test selection can also be affected by non-clinical factors such as physician familiarity or personal preference, local availability, and insurance coverage. The extent to which these factors impact test selection is not fully understood, as there is limited data on the effect of imaging modality selection on downstream resource use. This study sought to understand key differences between patient populations in the US receiving stress echocardiography, SPECT MPI, PET MPI and cCTA, and physician referral patterns for additional imaging or diagnostic tests and/or coronary interventional procedures.

## Methods

### Overview

This was a real-world, retrospective study of de-identified claims and electronic health records (EHR) data from the commercially available Decision Resources Group Real-World Evidence Data Repository US database (Clarivate), which sought to assess utilisation of imaging modalities and physician referral patterns. The database links medical claims, prescription claims and EHRs from government and commercial insurance plans for more than 300 million covered lives to provide longitudinal patient-level data, which includes tests ordered, test results, diagnoses, comorbidities, medications, therapies, and patient demographics [21]. Claims and EHR data were linked by a US Health Insurance Portability and Accountability Act (HIPAA)-compliant encrypted participant key generated by a third party. Data from all sources are linked via a direct-matching algorithm and then de-identified at the patient level, enabling longitudinal tracking of patients across data sources, sites of care (inpatient, outpatient, primary, specialty, etc.) and pharmacies. The repository represents more than 98% of US payers and is nationally representative of the US, including patients from all geographic regions. The database has been applied in multiple peer-reviewed real-world retrospective analyses [22–25].

### Patient population

**Fig. 1:**
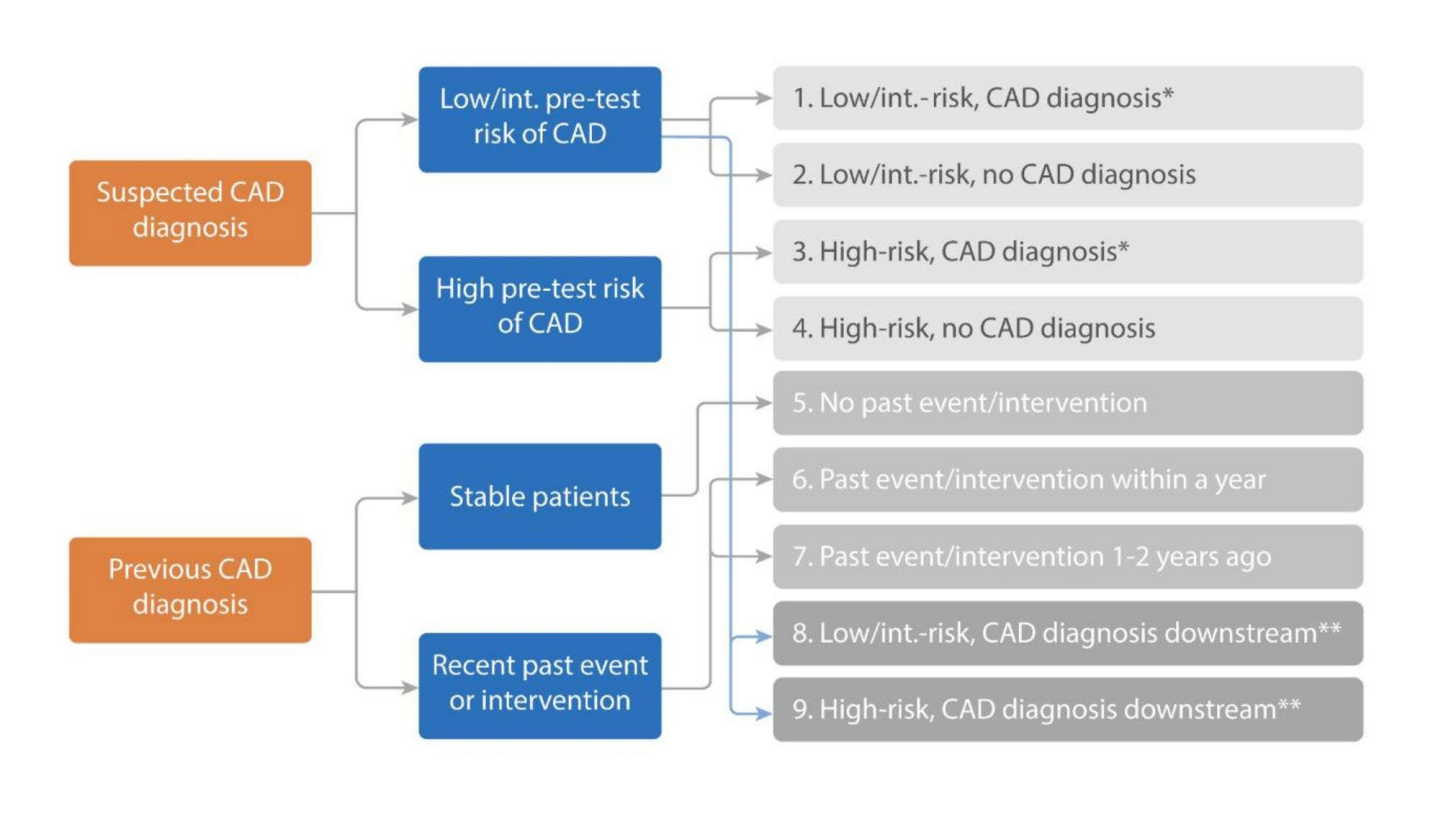
Stratification of patients. Abbreviations: CAD, coronary artery disease. Note: *CAD diagnosis following index (reference) test; **CAD diagnosis more than 3 months following index test.

Data were obtained for 2.5 million patients who received a stress echocardiography, SPECT MPI, PET MPI, or cCTA test at an index date (the earliest date for the imaging test) between January 2016 and March 2018. For inclusion, patients were required to have at least two claims within a 1-year and 2-year period pre-index, and at least two claims within a 1-year period post-index. Patients were not included if the coefficient of variation (CV) for the number of claims (calculated as the sum of the standard deviation [SD] and mean for the sample, divided by the mean) was greater than the sum of the SD and mean for the sample. Using diagnostic codes in the claims data, patients were stratified into two groups: patients with suspected CAD (Cohorts 1–4, 8 & 9) and those with an existing CAD diagnosis (Cohorts 5–7) at the index test (Fig. 1). Patients with suspected CAD were further segmented by pre-test risk and absence or presence of a CAD diagnosis within 3 months (Cohorts 1–4) or more than 3 months following the index test (Cohorts 8 & 9). As claims-based data does not capture symptoms of chest pain, a proxy pre-test CAD risk stratification method was used to stratify low/intermediate risk from high-risk patients based on sex, age, and underlying conditions (including diabetes, hypertension, and hyperlipidaemia) (Additional File Fig. 1) [26]. Patients with a previous CAD diagnosis (derived from diagnostic codes in the claims data) were stratified based on the presence and recency of acute cardiac events (acute coronary syndrome [ACS], unstable angina [UA], ST-elevation myocardial infarction [STEMI], non ST-elevation myocardial infarction [NSTEMI], ischaemic stroke [IS], transient ischaemic event [TIA], acute heart failure [AHF]) and/or interventions (percutaneous coronary intervention [PCI], coronary artery bypass graft [CABG]).

### Statistical analyses

In total, patients were stratified into nine cohorts (Fig. 1). The number of patients in each test segment were identified and matched based on age, sex, heart failure and atrial fibrillation/flutter/other cardiac arrhythmias. Patient groups were comparable in age, sex, and other variables, with a 1:1 male to female ratio, allowing for a fair comparison. Because many group comparison tests were performed, statistical variance can vary. A p-value cut-off of 0.05 was therefore not considered sufficient in this analysis, and so a more stringent cut-off of 0.005 was used. Linear regression was used to compare numeric variables (such as count of events and interventions), and logistic regression was used to compare categorical variables (such as presence of events and interventions). Differences in occurrence of several utilisation variables (including subsequent invasive and non-invasive diagnostic testing, interventions, and acute events) following use of different imaging modalities within each patient cohort were evaluated, controlling for intracohort variations. Frequency of referrals for diagnostic testing in different patient population was estimated.

## Results

### Patient characteristics

**Fig. 2:**
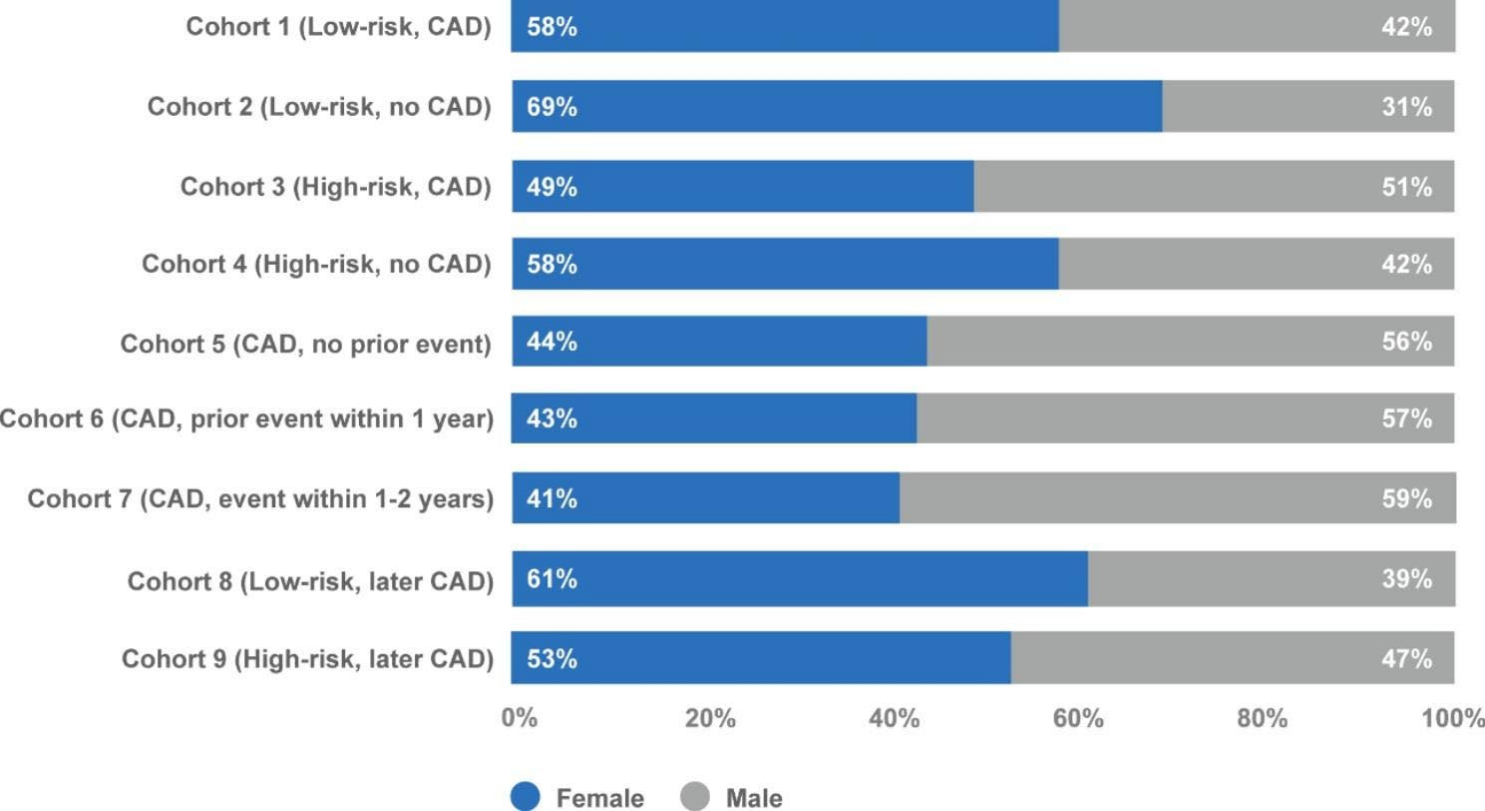
Sex distribution of patient population by cohort. Abbreviations: CAD, coronary artery disease

**Fig. 3:**
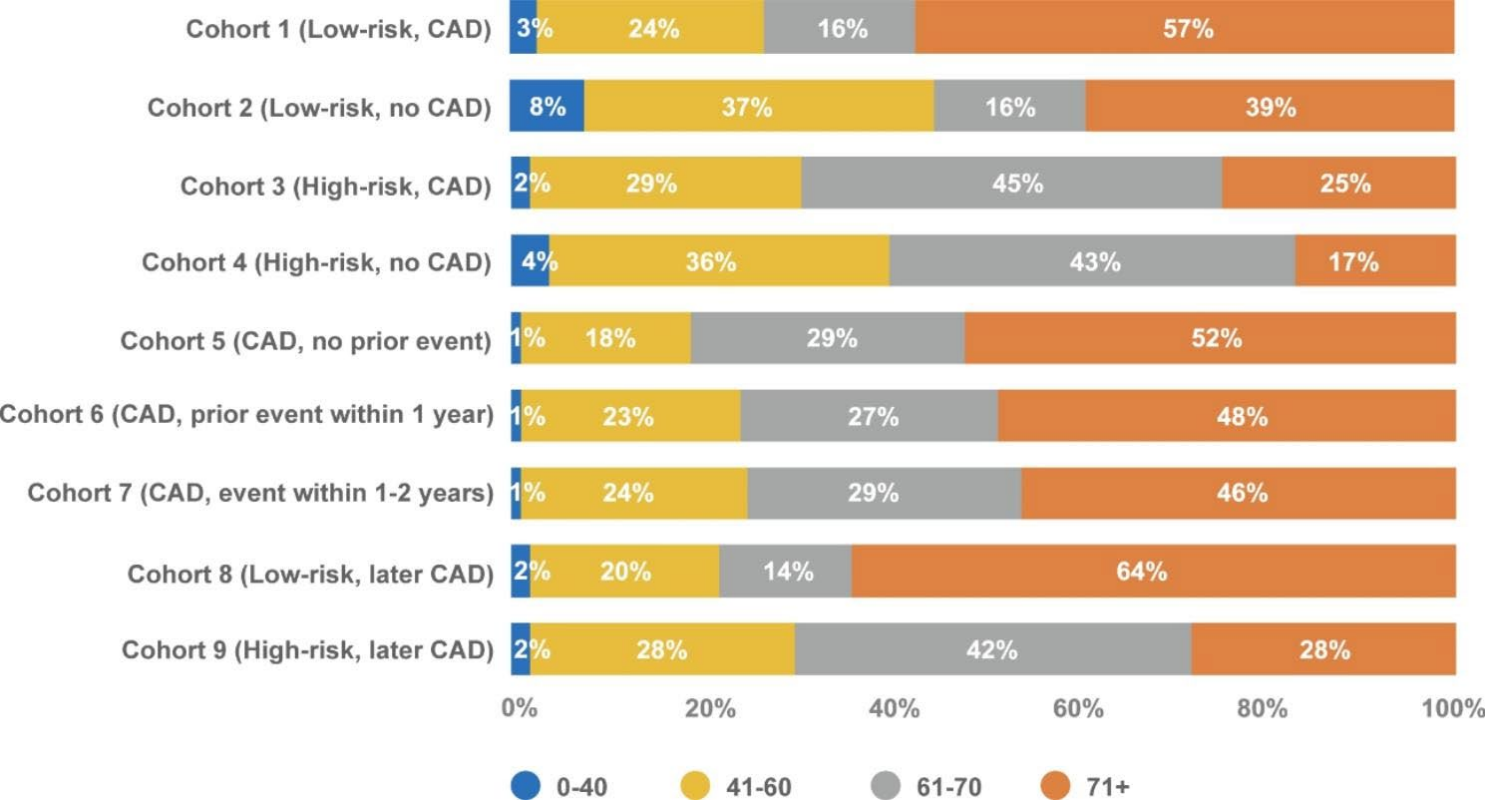
Age distribution of patient population by cohort. Abbreviations: CAD, coronary artery disease

Patient characteristics by cohort are presented in Figs. 2 and 3; Table 1. The overall sample of patients receiving CAD imaging tests included a higher proportion of older males. Of all the cohorts, the cohort of patients with a CAD diagnosis and events 1–2 years pre-index (Cohort 7) included the highest proportion of males (59%). The cohort of low-risk patients with no CAD diagnosis (Cohort 2) included the highest proportion of females (69%). Low-risk patients who later had a CAD diagnosis (Cohort 8) had the highest proportion of patients aged ≥ 70 (64%).

**Table 1:** Patient comorbidity profile by cohort

Cohort	Class 1–3 obesity	Diabetes	Symptoms and signs involving the circulatory and respiratory systems	Hypertensive diseases	Disorders of lipoprotein metabolism and other lipidemias	Other forms of heart disease	Gastro oesophageal reflux disease	Abnormal findings on examination of blood without diagnosis
**Cohort 1 (low pre-test risk, CAD diagnosis within 3 months of index test)**	43%	20%	83%	66%	59%	45%	33%	29%
**Cohort 2 (low pre-test risk, no CAD diagnosis following index test)**	43%	14%	87%	56%	51%	39%	33%	28%
**Cohort 3 (high pre-test risk, CAD diagnosis within 3 months of index test)**	60%	51%	88%	92%	85%	50%	40%	39%
**Cohort 4 (high pre-test risk, no CAD diagnosis following index test)**	61%	46%	89%	89%	82%	43%	42%	39%
**Cohort 5 (existing CAD diagnosis, no prior cardiac events)**	52%	44%	88%	92%	89%	64%	42%	36%
**Cohort 6 (existing CAD diagnosis, prior cardiac event within 1 year)**	52%	54%	97%	96%	91%	82%	48%	49%
**Cohort 7 (existing CAD diagnosis, prior cardiac event within 1–2 years)**	52%	51%	96%	96%	93%	80%	46%	44%
**Cohort 8 (low pre-test risk, subsequent CAD diagnosis more than 3 months after index test)**	42%	22%	88%	92%	89%	64%	42%	36%
**Cohort 9 (high pre-test risk, subsequent CAD diagnosis more than 3 months after index test)**	60%	51%	97%	96%	91%	82%	48%	49%

Abbreviations: CAD, coronary artery disease

As expected, based on the pre-test risk stratification method, high pre-test risk patients had a greater prevalence of comorbidities including diabetes, hypertensive disease, lipoprotein disorders, gastro oesophageal reflux disease and other non-ischaemic cardiac diseases (Table 1). Prevalence of diabetes ranged from 14% (Cohort 2) to 54% (Cohort 6), with high pre-test risk patients and those with an existing CAD diagnosis at least twice as likely to have the condition than low pre-test risk patients. Prevalence of obesity (Class 1–3) ranged from 42% (Cohort 8) to 61% (Cohort 4), with high pre-test risk patients and those with an existing CAD diagnosis more likely to be obese than low pre-test risk patients (Table 1 and Additional File Table 1). Patients with CAD with prior events were more symptomatic and had more comorbidities than patients without prior events on record.

Assessing comorbidities at the aggregated imaging level showed that patients who underwent stress echocardiography or cCTA had similar comorbidity profiles (Table 2). Separately, patients who underwent SPECT MPI and PET MPI also shared common comorbidity characteristics. Patients who underwent stress echocardiography and cCTA had a lower prevalence of hypertensive disease and lipoprotein disorders compared with patients who received SPECT MPI and PET MPI. Prevalence of circulatory and respiratory symptoms and gastro oesophageal reflux disease were similar across all imaging test groups.

**Table 2:** Patient comorbidity profile by imaging test

	Stress echocardiography	cCTA	SPECT MPI	PET MPI
Symptoms and signs involving the circulatory and respiratory systems	86%	86%	88%	85%
Hypertensive diseases	66%	68%	84%	89%
Disorders of lipoprotein metabolism and other dyslipidaemias	66%	64%	78%	81%
Other forms of heart disease	38%	49%	55%	61%
Gastro oesophageal reflux disease	35%	37%	40%	39%
Abnormal findings on examination of blood without diagnosis	33%	34%	35%	35%

Abbreviations: cCTA, coronary computed tomography angiography; PET MPI positron emission tomography, myocardial perfusion imaging; SPECT MPI, single-photon emission computed tomography MPI; stress echo, stress echocardiography

Among patients with an existing CAD diagnosis and prior cardiac events or interventions at the index test, the most common prior events/interventions were UA, NSTEMI, and PCI (Additional File Table 2). Those who underwent stress echocardiography were more likely to have had a STEMI or PCI, whereas patients who underwent more advanced imaging tests like PET MPI or cCTA were more likely to have experienced acute heart failure.

### Referral patterns

**Fig. 4:**
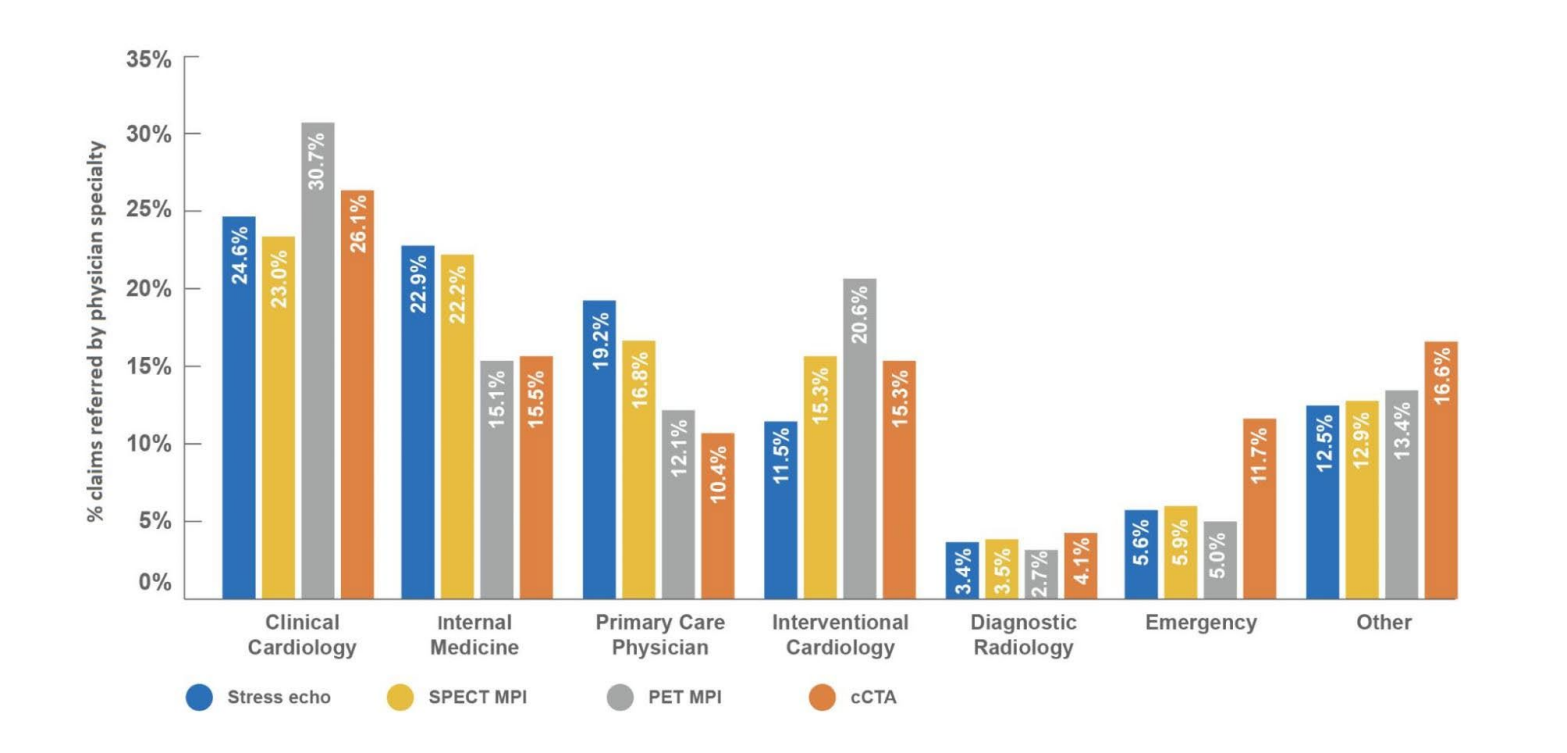
Referring physician distribution by index test. Abbreviations: cCTA, coronary computed tomography angiography; PET MPI, positron emission tomography myocardial perfusion imaging; SPECT MPI, single-photon emission computed tomography MPI; stress echo, stress echocardiography

Overall, 77% of patients received standalone SPECT MPI, and 18% underwent standalone stress echocardiography (Additional File Fig. 2). In contrast, utilisation of PET MPI and cCTA was relatively low, at 3.2% and 2.1%, respectively. Overall, 43% of physicians were single-test referrers for SPECT MPI, meaning that they referred more than 90% of their patients to that test. By contrast, the proportion of physicians who referred more than 90% of their patients to stress echocardiography, PET MPI or cCTA was just 3%, 1% and 1%, respectively. Cardiologists and interventional cardiologists were more likely to refer to PET MPI and cCTA, while family medicine and internal medicine physicians showed higher referral to stress echocardiography and SPECT MPI (Fig. 4 and Additional File Table 3). Emergency medicine physicians were more likely to refer patients to cCTA.

**Fig. 5:**
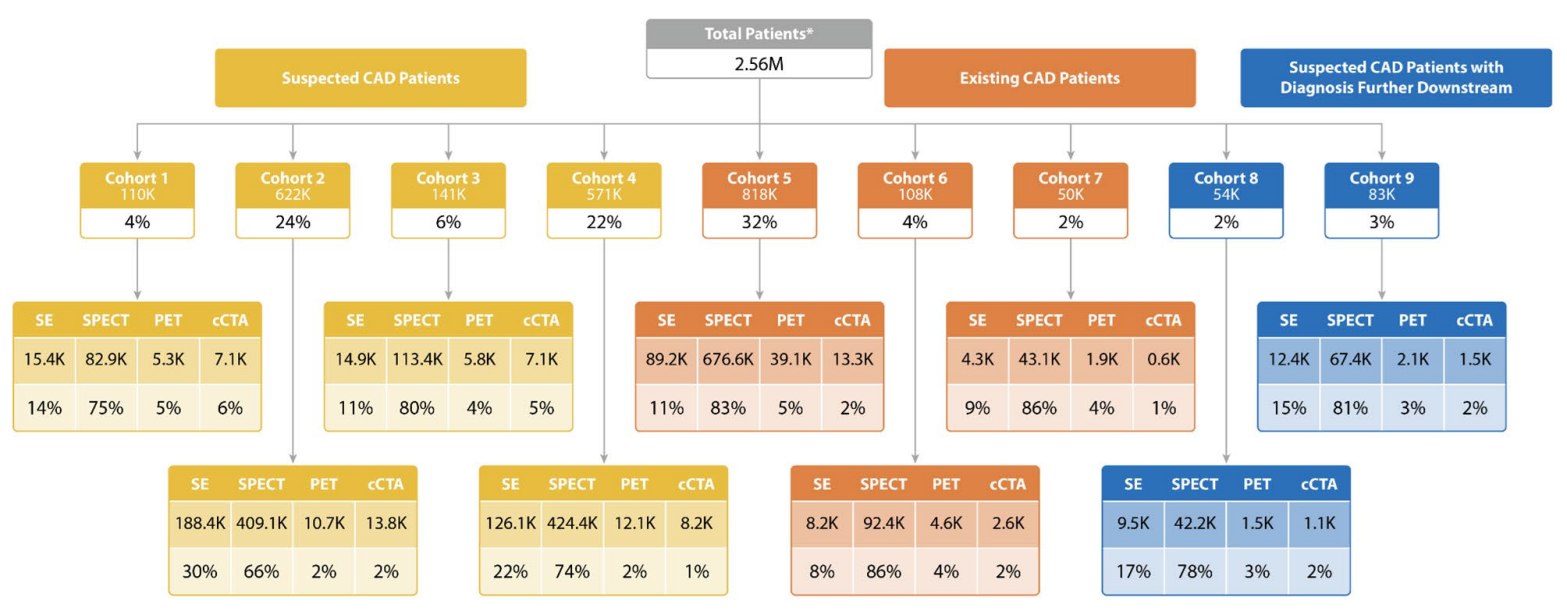
Patients receiving standalone imaging. Note: Cohort 1, low-risk with CAD; Cohort 2, low-risk without CAD; Cohort 3, high-risk with CAD; Cohort 4, high-risk without CAD; Cohort 5, CAD with no prior event; Cohort 6, CAD with prior event within 1 year; Cohort 7, CAD with event within 1–2 years; Cohort 8, low-risk with later CAD diagnosis; Cohort 9, high-risk with later CAD diagnosis. Abbreviations: CAD, coronary artery disease; cCTA, coronary computed tomography angiography; PET MPI, positron emission tomography myocardial perfusion imaging; SPECT MPI, single-photon emission computed tomography myocardial perfusion imaging; SE, stress echocardiography

**Fig. 6:**
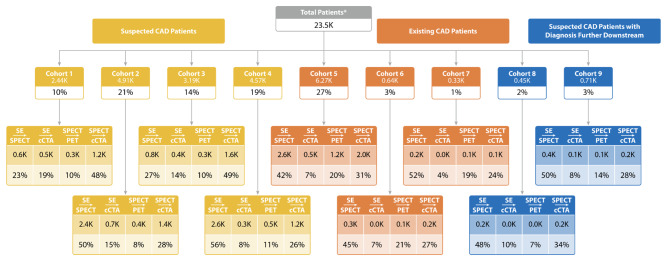
Patients with one of the following test combinations. Note: Cohort 1, low-risk with CAD; Cohort 2, low-risk without CAD; Cohort 3, high-risk with CAD; Cohort 4, high-risk without CAD; Cohort 5, CAD with no prior event; Cohort 6, CAD with prior event within 1 year; Cohort 7, CAD with event within 1–2 years; Cohort 8, low-risk with later CAD diagnosis; Cohort 9, high-risk with later CAD diagnosis. Abbreviations: CAD, coronary artery disease; cCTA, coronary computed tomography angiography; PET MPI, positron emission tomography myocardial perfusion imaging; SPECT MPI, single-photon emission computed tomography myocardial perfusion imaging; SE, stress echocardiography

The majority of patients who received a standalone primary imaging test did not have CAD (24% and 22% for Cohorts 2 and 4, respectively) or were patients with CAD with no prior events or interventions (32% for Cohort 5) (Fig. 5). As expected, patients receiving combinations of two imaging tests within 3 months were more likely to be diagnosed with CAD compared with patients receiving standalone tests (Fig. 6).

### Additional downstream imaging

Overall, aggregating all cohorts, a higher proportion of patients who underwent cCTA (6.6%) at the index date received additional imaging tests (SPECT MPI, PET MPI, cCTA, and stress echocardiography) compared with those who underwent SPECT MPI (3.4%), PET MPI (2.2%) or stress echocardiography (2.9%). Among patients with suspected CAD (Cohorts 1–4), follow-up imaging was uncommon (Additional File Table 4). In general, more than 90% of patients with suspected CAD did not receive a follow-up imaging test within 1 year (stress echocardiography, 93–97%; SPECT MPI, 93–97%; PET MPI, 94–97%; cCTA, 86–95%) (Additional File Table 4). High pre-test risk patients (Cohorts 3 and 4) were only marginally more likely to receive additional imaging compared with low/intermediate pre-test risk patients (Cohorts 1 and 2) (1–14% vs. 1–12%) (Additional File Table 4).

In patients with existing CAD and prior cardiac events in the past 2 years (Cohorts 6 [event in past 1 year] and 7 [event 1–2 years ago]), the rate of near-term (within 3 months) follow-up imaging was no greater than 10% (stress echocardiography, 1–2%; SPECT MPI, 3%; PET MPI, 2–3%; cCTA, 7–10%) (Additional File Table 4). Similarly, in patients with existing CAD and no prior cardiac events on record (Cohort 5), the likelihood of experiencing a downstream imaging test within 3 months was substantially increased in patients who underwent cCTA at the index date (stress echocardiography, 1%; SPECT MPI, 2%; PET MPI, 1%; cCTA, 7%). Due to a pre-test CAD diagnosis, the imaging tests experienced by this cohort are likely due to a change in symptoms and the need for additional disease characterisation information.

### Downstream coronary interventional procedures

Overall, a higher proportion of patients who underwent PET MPI (12.8%), SPECT MPI (12.4%) or cCTA (10.9%) at the index date received subsequent ICA compared with those who underwent stress echocardiography (6.0%). As expected, patients with suspected CAD who received a diagnosis up to 3 months following imaging (Cohorts 1 and 3) were substantially more likely to receive ICA than those without a diagnosis (Cohorts 2 and 4) (15–38% vs. 1–4%) (Additional File Table 5). Among low pre-test risk patients diagnosed with CAD within 3 months of their index imaging test (Cohort 1), 31% and 31% of patients who underwent SPECT MPI or stress echocardiography, respectively, subsequently underwent ICA. In contrast, just 17% and 15% of patients who underwent cCTA or PET MPI, respectively, at the index test subsequently had an ICA. Similar results were observed for high pre-test risk patients (Cohort 3), with 38%, 37%, 24% and 22% of patients who underwent SPECT MPI, stress echocardiography, PET MPI or cCTA, respectively, receiving downstream ICA.

## Discussion

This large retrospective real-world study of US claims and EHR data assessed key differences between patient populations receiving stress echocardiography, SPECT MPI, PET MPI and cCTA, and subsequent physician referrals to additional imaging or diagnostic tests and/or coronary interventional procedures. The results show that a higher proportion of patients underwent SPECT MPI compared with other imaging modalities, with proportionally very few patients undergoing PET MPI or cCTA. Most patients did not undergo follow-up imaging; in general, less than 10% of patients received a follow-up imaging test within 1 year. However, patients who underwent cCTA at the index date were more likely to subsequently undergo additional imaging tests compared with patients who underwent other imaging modalities.

At the aggregated imaging level, patients who received stress echocardiography or cCTA had similar comorbidity profiles. Patients who received SPECT MPI and PET MPI also shared similar comorbidity profiles, with these populations experiencing a greater comorbidity burden. As such, these patients are likely to have more frequent hospital visits and service utilisation [27]. Patients who received more advanced imaging tests such as PET MPI or cCTA were more likely to have experienced acute heart failure prior to the index test, whereas patients who received stress echocardiography were more likely to have experienced STEMI or PCI.

Analysis of physician referrals for various CAD imaging techniques suggests physicians are more likely to refer patients to SPECT MPI. The overall high use of SPECT MPI across patients of different disease states or severity indicates that physicians may be selecting imaging modalities based on local availability and expertise rather than clinical factors. Evidence suggests that SPECT MPI is more widely used due to affordability and availability [28, 29]. PET MPI is generally only available in tertiary hospitals, while cCTA is widely available in specialist referral centres. Physicians who refer patients for single tests are likely practicing in community settings or may be providers of those specific tests (for instance, clinics with SPECT MPI facilities).

When referring patients for imaging tests, an additional risk for physicians to consider is the radiation exposure to patients. The estimated whole-body effective radiation dose that a patient is exposed to is directly related to the half-life and the dose of the radiotracer that is administered [30]. SPECT MPI provides a higher radiation dose to patients compared with cCTA and PET MPI [31]. However, if downstream testing is required post cCTA, patients will be exposed to an overall higher dose of radiation. Radiation exposure can be minimised by following state-of-the-art practices and using modern instrumentation which operate at lower doses. To avoid unnecessary radiation exposure, it is important to individualise the choice of imaging modalities to the patient. However, risks related to radiation exposure must also be considered within the context of the potential benefit that the imaging modality may provide to the patient [32].

Generally, follow-up imaging was uncommon. However, patients who underwent cCTA were more likely to undergo additional imaging tests compared with those who received other imaging modalities. This analysis found that, while referrals to cCTA were low overall, emergency medicine physicians referred most of their patients for cCTA. cCTA is widely perceived as a convenient and accurate imaging modality, and is a key focus of the 2021 AHA/ACC/ASE/CHEST/SAEM/SCCT/SCMR Guideline for the Evaluation and Diagnosis of Chest Pain [33]. cCTA works well as a rule out test and is predominantly used in emergency departments for low-risk patients. However, in high-risk patients, cCTA is less useful and is susceptible to false positives [34]. The findings in this analysis are consistent with the ROMICAT-II study, a multicentre trial which reported that utilisation of cCTA in emergency departments improved the efficiency of clinical decision making compared with standard care [35]. However, incorporating cCTA also increased the number of revascularisation procedures, downstream testing and radiation exposure without any reduction in mortality or overall healthcare costs [35].

Among patients with suspected CAD who received a diagnosis up to 3 months following imaging (Cohorts 1 and 3), those evaluated using SPECT MPI and stress echocardiography were more likely to be recommended for an ICA in their first year of diagnosis compared with patients that underwent PET MPI and cCTA. Several factors can account for these findings. For instance, higher rates of ICA could be related to the lower sensitivity of SPECT MPI and stress echocardiography [28]. The high negative predictive value of PET MPI is sufficient to identify severe CAD, such that unnecessary ICA can be avoided in patients with non-significant reversible perfusion defects [36]. In addition, the relatively low rates of ICA in those who underwent cCTA could be in part attributable to the fact that additional imaging was more frequent in that group, eliminating the need for ICA.

Compared with other specialties studied in this analysis, cardiology departments referred the most patients for imaging tests, and were most likely to recommend PET MPI. PET MPI is considered a more advanced modality compared with SPECT MPI, is generally only available in tertiary hospitals, and is likely more accessible to cardiologists compared with other specialists [37]. As such, cardiologists are more likely to be aware of PET MPI and its benefits. Additionally, patients treated by cardiologists are more likely to be at greater risk of CAD than those treated by other healthcare professionals and are more likely to benefit from PET MPI than lower-risk patients [37, 38].

As the choice of imaging test determines downstream healthcare utilisation, the selection of imaging tests based on considerations other than clinical factors may be suboptimal. The 2021 AHA/ACC/ASE/CHEST/SAEM/SCCT/SCMR Guideline for the Evaluation and Diagnosis of Chest Pain acknowledges that testing choice will be influenced by site expertise, availability, and cost [33]. This may lead to unnecessary hospitalisations and increased costs for healthcare systems. In addition, individuals may be subjected to avoidable invasive techniques which are associated with low but non-negligeable risks such as local vascular injuries, conduction disturbances and contrast induced nephropathy.

Though ICA is the gold standard for diagnosing obstructive CAD, non-invasive imaging modalities provide functional and anatomical definition of atherosclerosis, subsequently guiding post-imaging therapeutic choices [10]. The capacity to identify unstable plaques is limited with ICA, and underestimates disease severity in people with diffuse coronary involvement [10]. ICA may be more appropriate when an interventional procedure is forecasted after CAD has been diagnosed using a non-invasive imaging technique [10]. Available evidence has led to a general recommendation that ICA with PCI should only be used in patients whose symptoms are resistant to optimal medical therapy or those in whom prognostically significant disease is likely [39]. This strengthens the role of non-invasive imaging modalities in determining prognosis, guiding patient selection for ICA and appropriate therapies, while providing cost-effective care. Further research is needed to understand factors that determine the selection of imaging modalities and how they impact downstream healthcare utilisation.

Like other analyses of this type, this study has limitations. Although there are systems in place to ensure data quality, real-world data obtained from routine clinical practice are prone to missing and erroneous data, coding imperfections, a lack of standardisation of clinical measures, variations between clinical testing centres, and measurements that are taken with varying periodicity. Furthermore, certain covariates of interest (e.g. comorbidities) may not be recorded consistently in the database. In addition, this study was conducted on a US population and therefore may not be generalisable to other populations as different trends are observed in different countries. A recent study of patients in England demonstrated that there is a sustained and consistent increase in the use of imaging investigations for CAD, with substantial shifts in the use of specific individual imaging modalities [40]. Use of cCTA increased, and greater regional increases in CCTA were associated with fewer hospitalisations for myocardial infarction and a more rapid decline in CAD mortality. Modest reductions in ICA were reported [40]. However, the data presented for the current, US-based study provide valuable evidence for decision makers to understand key characteristics of patient population with CAD and its treatment implications.

## Conclusion

This retrospective analysis of US real-world claims data demonstrated that physicians are more likely to recommend SPECT MPI for CAD risk evaluation compared with other non-invasive imaging modalities. Patients who underwent cCTA at the index date were more likely to undergo additional imaging tests compared with those who underwent other imaging modalities. To maximise the role of non-invasive imaging modalities in CAD patient management, further research is needed to understand the factors determining physician referral patterns.

## Electronic supplementary material

Below is the link to the electronic supplementary material.

Additional File: Characteristics and key differences between patient populations receiving imaging modalities for coronary artery disease diagnosis in the US: Supplemental appendix

## Data Availability

The data that support the findings of this study are available from Clarivate but restrictions apply to the availability of these data, which were used under license for the current study, and so are not publicly available. Data are however available from the authors upon reasonable request and with permission of Clarivate.

## Abbreviations

CAD: Coronary artery disease
ICA: Invasive coronary angiography
MPI: Myocardial perfusion imaging
SPECT: Single-photon emission computed tomography
PET: Positron emission tomography
PCI: Percutaneous coronary intervention
cCTA: Coronary computed tomography angiography
ACS: Acute coronary syndrome
UA: Unstable angina
STEMI: ST-elevation myocardial infarction
NSTEM: Non ST-elevation myocardial infarction
IS: Ischaemic stroke
TIA: Transient ischaemic event
AHF: Acute heart failure
CABG: Coronary artery bypass graft
