# Supplementary material for: Characteristics and key differences between patient populations receiving imaging modalities for coronary artery disease diagnosis in the US
Source: BMC Cardiovasc Disord. 2023 May 15;23:251. doi: 10.1186/s12872-023-03218-7 (PMC10186749; doi:10.1186/s12872-023-03218-7)
Supplement: Supplementary file 1 — Additional File: Characteristics and key differences between patient populations receiving imaging modalities for coronary artery disease diagnosis in the US: Supplemental appendix [file 12872_2023_3218_MOESM1_ESM.docx]

**Characteristics and key differences between patient populations receiving imaging modalities for coronary artery disease diagnosis in the US: Supplemental appendix**

# **Contents**

[Table S1: Distribution of obesity by patient cohort 3](#_Toc128482012)

[Table S2: Prior events and interventions in patients with existing CAD by index imaging modality 4](#_Toc128482013)

[Table S3: Physician referral patterns 6](#_Toc128482014)

[Table S4: Proportion of patients receiving follow-up imaging by cohort 12](#_Toc128482015)

[Table S5: Proportion of patients receiving downstream coronary angiography, by index test 17](#_Toc128482016)

[Figure S1: Proxy pre-test CAD risk stratification method 2](#_Toc125711682)

[Figure S2: Proportion of patients receiving a standalone test 5](#_Toc125711683)

#### Figure S1: Proxy pre-test CAD risk stratification method


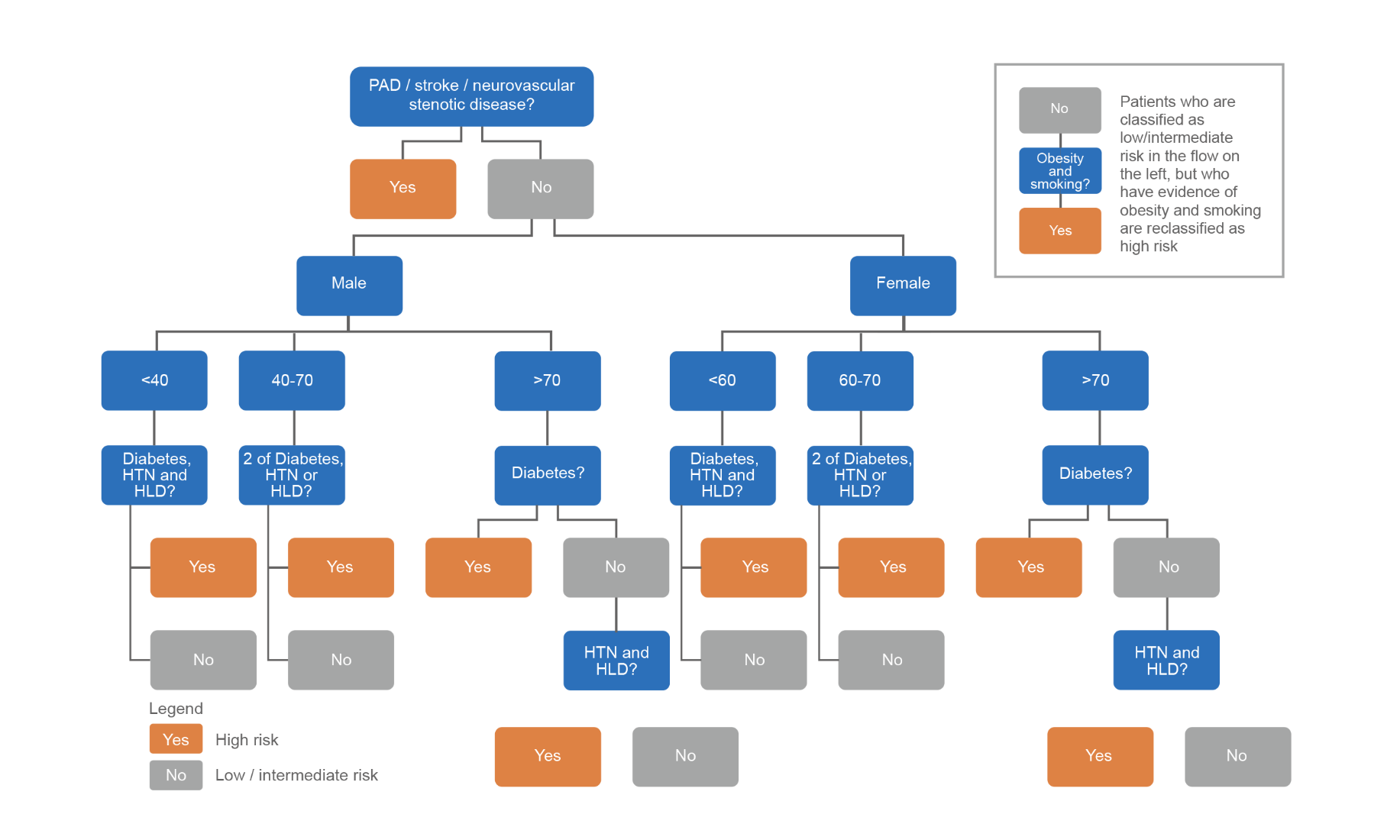


Abbreviations: CAD, coronary artery disease; HLD, hyperlipidaemia; HTN, hypertension; PAD, peripheral arterial disease.

#### Table S1: Distribution of obesity by patient cohort

| **Cohort** | **Class 1 obesity** | **Class 2 obesity** | **Class 3 obesity** | **Aggregate across all obesity classes** | **Normal weight** | **Overweight** | **Underweight** |
| --- | --- | --- | --- | --- | --- | --- | --- |
| **Cohort 1 (low pre-test risk, CAD diagnosis within 3 months of index test)** | 24% | 12% | 7% | 43% | 21% | 34% | 2% |
| **Cohort 2 (low pre-test risk, no CAD diagnosis following index test)** | 23% | 12% | 8% | 43% | 21% | 33% | 3% |
| **Cohort 3 (high pre-test risk, CAD diagnosis within 3 months of index test)** | 28% | 18% | 14% | 60% | 12% | 26% | 2% |
| **Cohort 4 (high pre-test risk, no CAD diagnosis following index test)** | 27% | 19% | 15% | 61% | 12% | 26% | 2% |
| **Cohort 5 (existing CAD diagnosis, no prior cardiac events)** | 27% | 15% | 10% | 52% | 15% | 31% | 2% |
| **Cohort 6 (existing CAD diagnosis, prior cardiac event within 1 year)** | 27% | 16% | 10% | 52% | 15% | 30% | 2% |
| **Cohort 7 (existing CAD diagnosis, prior cardiac event within 1–2 years)** | 27% | 16% | 10% | 52% | 16% | 30% | 2% |
| **Cohort 8 (low pre-test risk, subsequent CAD diagnosis more than 3 months after index test)** | 24% | 12% | 7% | 42% | 21% | 34% | 3% |
| **Cohort 9 (high pre-test risk, subsequent CAD diagnosis more than 3 months after index test)** | 28% | 18% | 14% | 60% | 12% | 26% | 2% |

Abbreviations: CAD, coronary artery disease.

#### Table S2: Prior events and interventions in patients with existing CAD by index imaging modality

| **Cohort** | **UA** | **STEMI** | **NSTEMI** | **IS** | **TIA** | **AHF** | **PCI** | **CABG** |
| --- | --- | --- | --- | --- | --- | --- | --- | --- |
| **Cohort 6 – existing CAD diagnosis, prior cardiac event within 1 year** | | | | | | | | |
| **Stress echocardiography** | 36% | 22% | 31% | 16% | 5% | 18% | 36% | 3% |
| **SPECT MPI** | 33% | 16% | 31% | 20% | 4% | 25% | 27% | 2% |
| **PET MPI** | 29% | 15% | 30% | 19% | 3% | 34% | 26% | 3% |
| **cCTA** | 32% | 12% | 34% | 17% | 3% | 29% | 20% | 2% |
| **Cohort 7 – existing CAD diagnosis, prior cardiac event within 1–2 years** | | | | | | | | |
| **Stress echocardiography** | 28% | 25% | 29% | 17% | 9% | 10% | 35% | 4% |
| **SPECT MPI** | 28% | 20% | 27% | 20% | 10% | 14% | 31% | 4% |
| **PET MPI** | 26% | 14% | 23% | 23% | 9% | 19% | 26% | 4% |
| **cCTA** | 24% | 10% | 20% | 27% | 12% | 21% | 15% | 2% |

Abbreviations: AHF, acute heart failure; CABG, coronay bypass graft; CAD, coronary artery disease; cCTA, coronary computed tomography angiography; NSTEMI, IS, ischaemic stroke; non-ST-elevation myocardial infarction; PCI, percutaneous coronary intervention; PET MPI, positron emission tomography myocardial perfusion imaging; SPECT MPI, single-photon emission computed tomography myocardial perfusion imaging; STEMI, ST-elevation myocardial infarction; TIA, transient ischaemic event; UA, unstable angina.

#### Figure S2: Proportion of patients receiving a standalone test


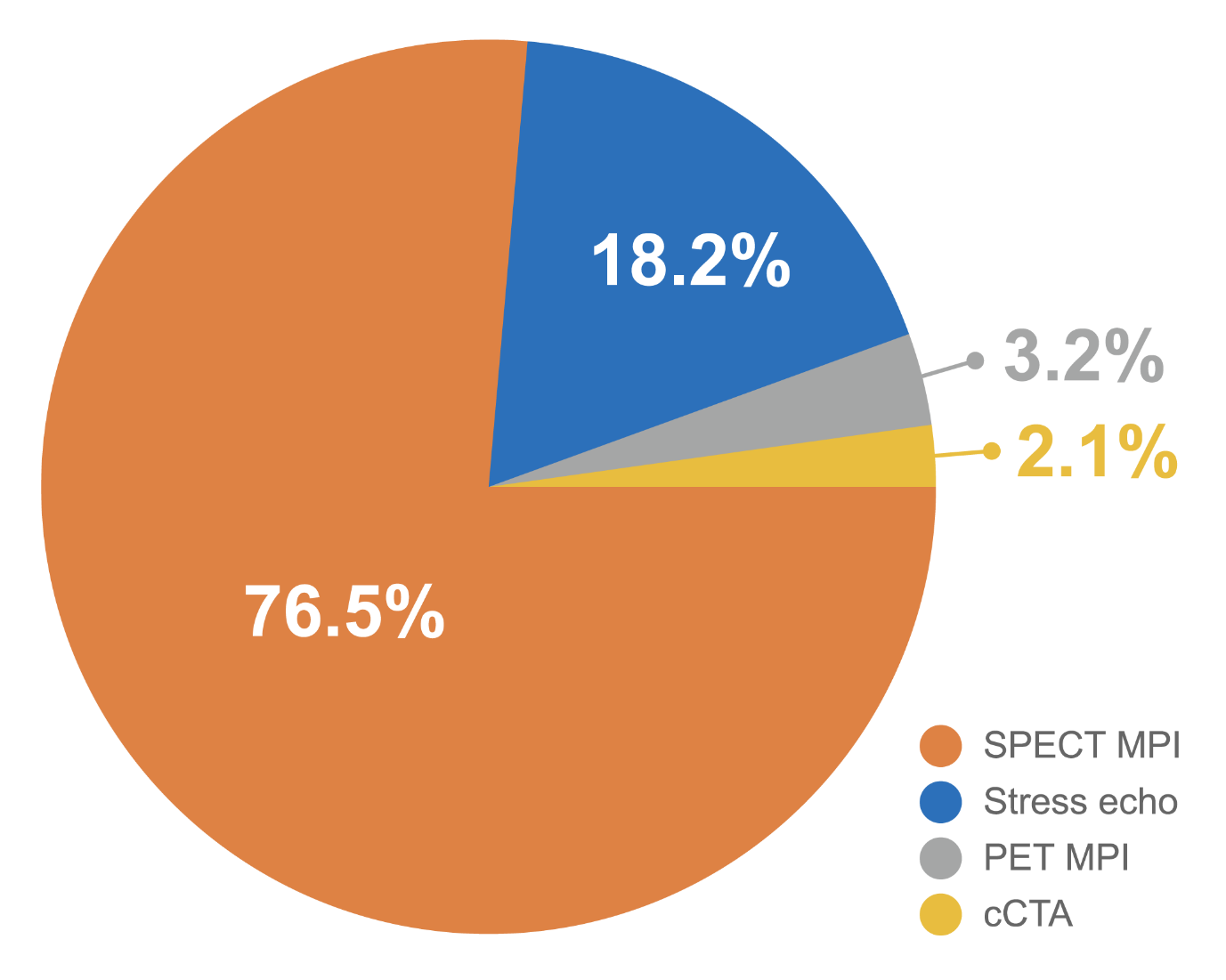


Abbreviations: cCTA, coronary computed tomography angiography; PET MPI, positron emission tomography myocardial perfusion imaging; SPECT MPI, single-photon emission computed tomography MPI; stress echo, stress echocardiography.

#### Table S3: Physician referral patterns

|  | **Stress echocardiography** | | | | **SPECT MPI** | | | **PET MPI** | | | **cCTA** | | |
| --- | --- | --- | --- | --- | --- | --- | --- | --- | --- | --- | --- | --- | --- |
| **Cohort 1 – low pre-test risk, CAD diagnosis within 3 months of index test** | | | | | | | | | | | | | |
| Index test referring physician | Cardiovascular disease | 25% | | | Cardiovascular disease | | 25% | Cardiovascular disease | 34% | Cardiovascular disease | | 27% | |
|  | Internal medicine | 24% | | | Internal medicine | | 22% | Internal medicine | 15% | Internal medicine | | 16% | |
|  | Family medicine | 14% | | | Family medicine | | 13% | Interventional cardiology | 14% | Interventional cardiology | | 9% | |
|  | Interventional cardiology | 7% | | | Interventional cardiology | | 9% | Family medicine | 8% | Emergency medicine | | 8% | |
|  | Emergency medicine | 4% | | | Emergency medicine | | 4% | Specialist | 4% | Family medicine | | 7% | |
|  | Others | 27% | | | Others | | 27% | Others | 24% | Others | | 34% | |
| Downstream physician | Cardiovascular disease | 53% | | | Cardiovascular disease | | 54% | Internal medicine | 42% | Cardiovascular disease | | 46% | |
|  | Internal medicine | 47% | | | Internal medicine | | 48% | Cardiovascular disease | 41% | Internal medicine | | 46% | |
|  | Family medicine | 39% | | | Family medicine | | 43% | Family medicine | 38% | Family medicine | | 38% | |
|  | Diagnostic radiology | 27% | | | Diagnostic radiology | | 25% | Diagnostic radiology | 20% | Diagnostic radiology | | 32% | |
|  | Interventional cardiology | 22% | | | Interventional cardiology | | 23% | Emergency medicine | 19% | Emergency medicine | | 23% | |
| **Cohort 2 – low pre-test risk, no CAD diagnosis following index test** | | | | | | | | | | | | | |
| Index test referring physician | Cardiovascular disease | 24% | | | Cardiovascular disease | | 23% | Cardiovascular disease | 33% | | Cardiovascular disease | | 25% |
|  | Internal medicine | 22% | | | Internal medicine | | 21% | Internal medicine | 14% | | Emergency medicine | | 13% |
|  | Family medicine | 16% | | | Family medicine | | 14% | Interventional cardiology | 11% | | Internal medicine | | 13% |
|  | Interventional cardiology | 6% | | | Interventional cardiology | | 8% | Family medicine | 9% | | Interventional cardiology | | 7% |
|  | Emergency medicine | 5% | | | Emergency medicine | | 5% | Emergency medicine | 5% | | Family medicine | | 7% |
|  | Others | 28% | | | Others | | 29% | Others | 29% | | Others | | 35% |
|  | **Stress echocardiography** | | | | **SPECT MPI** | | | **PET MPI** | | | **cCTA** | | |
| Downstream physician | Family medicine | 48% | | | Family medicine | | 47% | Family medicine | 42% | | Family medicine | | 41% |
|  | Internal medicine | 42% | | | Internal medicine | | 43% | Internal medicine | 40% | | Internal medicine | | 39% |
|  | Diagnostic radiology | 33% | | | Cardiovascular disease | | 36% | Cardiovascular disease | 39% | | Diagnostic radiology | | 36% |
|  | Cardiovascular disease | 29% | | | Diagnostic radiology | | 29% | Diagnostic radiology | 24% | | Cardiovascular disease | | 31% |
|  | Emergency medicine | 19% | | | Emergency medicine | | 21% | Emergency medicine | 20% | | Emergency medicine | | 27% |
| **Cohort 3 – high pre-test risk, CAD diagnosis within 3 months of index test** | | | | | | | | | | | | | |
| Index test referring physician | Cardiovascular disease | 27% | | Cardiovascular disease | | 25% | | Cardiovascular disease | 32% | | Cardiovascular disease | | 27% |
|  | Internal medicine | 24% | | Internal medicine | | 23% | | Internal medicine | 17% | | Internal medicine | | 16% |
|  | Family medicine | 14% | | Family medicine | | 13% | | Interventional cardiology | 14% | | Emergency medicine | | 9% |
|  | Interventional cardiology | 6% | | Interventional cardiology | | 9% | | Family medicine | 8% | | Interventional cardiology | | 8% |
|  | Emergency medicine | 4% | | Emergency medicine | | 4% | | Specialist | 4% | | Family medicine | | 6% |
|  | Others | 25% | | Others | | 27% | | Others | 25% | | Others | | 33% |
| Downstream physician | Cardiovascular disease | 59% | Cardiovascular disease | | | 60% | | Cardiovascular disease | 51% | | Internal medicine | | 54% |
|  | Internal medicine | 53% | Internal medicine | | | 53% | | Internal medicine | 51% | | Cardiovascular disease | | 53% |
|  | Family medicine | 45% | Family medicine | | | 49% | | Family medicine | 48% | | Family medicine | | 44% |
|  | Diagnostic radiology | 32% | Diagnostic radiology | | | 30% | | Diagnostic radiology | 27% | | Diagnostic radiology | | 37% |
|  | Emergency medicine | 25% | Emergency medicine | | | 28% | | Emergency medicine | 25% | | Emergency medicine | | 31% |

|  | **Stress echocardiography** | | | | **SPECT MPI** | | **PET MPI** | | **cCTA** | | | |
| --- | --- | --- | --- | --- | --- | --- | --- | --- | --- | --- | --- | --- |
| **Cohort 4 – high pre-test risk, no CAD diagnosis following index test** | | | | | | | | | | | | |
| Index test referring physician | Cardiovascular disease | 24% | | Cardiovascular disease | | 23% | Cardiovascular disease | 33% | Cardiovascular disease | 25% | | |
|  | Internal medicine | 23% | | Internal medicine | | 22% | Internal medicine | 14% | Internal medicine | 15% | | |
|  | Family medicine | 16% | | Family medicine | | 14% | Interventional cardiology | 10% | Emergency medicine | 12% | | |
|  | Interventional cardiology | 6% | | Interventional cardiology | | 8% | Family medicine | 9% | Interventional cardiology | 7% | | |
|  | Emergency medicine | 5% | | Emergency medicine | | 5% | Emergency medicine | 4% | Family medicine | 7% | | |
|  | Other | 25% | | Other | | 28% | Other | 29% | Other | 34% | | |
| Downstream physician | Family medicine | 49% | | Family medicine | | 51% | Family medicine | 48% | Internal medicine | | 51% | |
|  | Internal medicine | 48% | | Internal medicine | | 49% | Internal medicine | 45% | Family medicine | | 44% | |
|  | Diagnostic radiology | 36% | | Cardiovascular disease | | 40% | Cardiovascular disease | 43% | Diagnostic radiology | | 41% | |
|  | Cardiovascular disease | 33% | | Diagnostic radiology | | 33% | Diagnostic radiology | 30% | Cardiovascular disease | | 37% | |
|  | Emergency medicine | 24% | | Emergency medicine | | 27% | Emergency medicine | 26% | Emergency medicine | | 35% | |
| **Cohort 5 – existing CAD diagnosis, no prior cardiac events** | | | | | | | | | | | | |
| Index test referring physician | Cardiovascular disease | 31% | | Cardiovascular disease | | 28% | Cardiovascular disease | 32% | Cardiovascular disease | | 28% | |
|  | Internal medicine | 20% | | Internal medicine | | 20% | Interventional cardiology | 14% | Internal medicine | | 14% | |
|  | Family medicine | 11% | | Family medicine | | 12% | Internal medicine | 14% | Interventional cardiology | | 11% | |
|  | Interventional cardiology | 10% | | Interventional medicine | | 11% | Family medicine | 10% | Emergency medicine | | 6% | |
|  | Emergency medicine | 3% | | Emergency medicine | | 4% | Emergency medicine | 3% | Family medicine | | 5% | |
|  | Other | 25% | | Other | | 26% | Other | 27% | Other | | 36% | |
|  | **Stress echocardiography** | | | | **SPECT MPI** | | **PET MPI** | | **cCTA** | | | |
| Downstream physician | Internal medicine | 51% | Cardiovascular disease | | | 55% | Cardiovascular disease | 52% | Cardiovascular disease | | 58% | |
|  | Cardiovascular disease | 50% | Internal medicine | | | 52% | Internal medicine | 48% | Internal medicine | | 54% | |
|  | Family medicine | 43% | Family medicine | | | 48% | Family medicine | 48% | Family medicine | | 42% | |
|  | Diagnostic radiology | 32% | Diagnostic radiology | | | 28% | Emergency medicine | 24% | Diagnostic radiology | | 37% | |
|  | Emergency medicine | 23% | Emergency medicine | | | 27% | Diagnostic radiology | 22% | Emergency medicine | | 30% | |
| **Cohort 6 – existing CAD diagnosis, prior cardiac event within 1 year** | | | | | | | | | | | | |
| Index test referring physician | Cardiovascular disease | 31% | Cardiovascular disease | | | 27% | Cardiovascular disease | 33% | Cardiovascular disease | | | 24% |
|  | Internal medicine | 20% | Internal medicine | | | 23% | Internal medicine | 16% | Internal medicine | | | 20% |
|  | Interventional cardiology | 12% | Interventional cardiology | | | 11% | Interventional cardiology | 13% | Interventional cardiology | | | 12% |
|  | Family medicine | 9% | Family medicine | | | 9% | Family medicine | 6% | Emergency medicine | | | 7% |
|  | Emergency medicine | 5% | Emergency medicine | | | 5% | Emergency medicine | 5% | Family medicine | | | 5% |
|  | Other | 23% | Other | | | 25% | Other | 26% | Other | | | 32% |
| Downstream physician | Cardiovascular disease | 66% | Cardiovascular disease | | | 65% | Cardiovascular disease | 69% | Cardiovascular disease | | | 73% |
|  | Internal medicine | 58% | Internal medicine | | | 64% | Internal medicine | 63% | Internal medicine | | | 67% |
|  | Family medicine | 47% | Family medicine | | | 53% | Family medicine | 52% | Family medicine | | | 48% |
|  | Emergency medicine | 40% | Emergency medicine | | | 46% | Emergency medicine | 45% | Diagnostic radiology | | | 46% |
|  | Diagnostic radiology | 37% | Diagnostic radiology | | | 36% | Diagnostic radiology | 36% | Emergency medicine | | | 46% |

|  | **Stress echocardiography** | | | | **SPECT MPI** | | **PET MPI** | | **cCTA** | |
| --- | --- | --- | --- | --- | --- | --- | --- | --- | --- | --- |
| **Cohort 7 – existing CAD diagnosis, prior cardiac event within 1–2 years** | | | | | | | | | | |
| Index test referring physician | Cardiovascular disease | 32% | | Cardiovascular disease | | 28% | Cardiovascular disease | 33% | Cardiovascular disease | 23% |
|  | Internal medicine | 18% | | Internal medicine | | 21% | Interventional cardiology | 17% | Internal medicine | 18% |
|  | Interventional cardiology | 13% | | Interventional cardiology | | 12% | Internal medicine | 14% | Interventional cardiology | 12% |
|  | Family medicine | 10% | | Family medicine | | 10% | Family medicine | 8% | Emergency medicine | 8% |
|  | Emergency medicine | 4% | | Emergency medicine | | 5% | Emergency medicine | 4% | Family medicine | 5% |
|  | Other | 23% | | Other | | 24% | Other | 25% | Other | 35% |
| Downstream physician | Cardiovascular disease | 58% | | Cardiovascular disease | | 61% | Cardiovascular disease | 80% | Cardiovascular disease | 67% |
|  | Internal medicine | 53% | | Internal medicine | | 57% | Internal medicine | 55% | Internal medicine | 67% |
|  | Family medicine | 46% | | Family medicine | | 50% | Family medicine | 49% | Emergency medicine | 47% |
|  | Emergency medicine | 34% | | Emergency medicine | | 39% | Emergency medicine | 37% | Family medicine | 46% |
|  | Diagnostic radiology | 33% | | Diagnostic radiology | | 32% | Interventional cardiology | 30% | Diagnostic radiology | 40% |
| **Cohort 8 – low pre-test risk, subsequent CAD diagnosis more than 3 months after index test** | | | | | | | | | | |
| Index test referring physician | Cardiovascular disease | 26% | | Cardiovascular disease | | 25% | Cardiovascular disease | 35% | Cardiovascular disease | 23% |
|  | Internal medicine | 23% | | Internal medicine | | 21% | Internal medicine | 14% | Internal medicine | 15% |
|  | Family medicine | 14% | | Family medicine | | 13% | Interventional cardiology | 12% | Emergency medicine | 10% |
|  | Interventional cardiology | 6% | | Interventional cardiology | | 9% | Family medicine | 6% | Interventional cardiology | 9% |
|  | Emergency medicine | 4% | | Emergency medicine | | 4% | Emergency medicine | 5% | Family medicine | 6% |
|  | Other | 27% | | Other | | 28% | Other | 29% | Other | 38% |
|  | **Stress echocardiography** | | | | **SPECT MPI** | | **PET MPI** | | **cCTA** | |
| Downstream physician | Internal medicine | 50% | | Cardiovascular disease | | 51% | Internal medicine | 48% | Internal medicine | 48% |
|  | Cardiovascular disease | 48% | | Internal medicine | | 51% | Cardiovascular disease | 48% | Cardiovascular disease | 44% |
|  | Family medicine | 43% | | Family medicine | | 47% | Family medicine | 41% | Family medicine | 39% |
|  | Diagnostic radiology | 35% | | Diagnostic radiology | | 30% | Emergency medicine | 28% | Diagnostic radiology | 36% |
|  | Emergency medicine | 25% | | Emergency medicine | | 28% | Diagnostic radiology | 24% | Emergency medicine | 31% |
| **Cohort 9 – high pre-test risk, subsequent CAD diagnosis more than 3 months after index test** | | | | | | | | | | |
| Index test referring physician | Cardiovascular disease | 26% | Cardiovascular disease | | | 24% | Cardiovascular disease | 36% | Cardiovascular disease | 25% |
|  | Internal medicine | 23% | Internal medicine | | | 22% | Internal medicine | 13% | Internal medicine | 14% |
|  | Family medicine | 14% | Family medicine | | | 12% | Interventional cardiology | 13% | Interventional cardiology | 10% |
|  | Interventional cardiology | 6% | Interventional cardiology | | | 8% | Family medicine | 7% | Emergency medicine | 9% |
|  | Emergency medicine | 4% | Emergency medicine | | | 5% | Emergency medicine | 4% | Family medicine | 8% |
|  | Other | 26% | Other | | | 28% | Other | 27% | Other | 35% |
| Downstream physician | Internal medicine | 56% | Internal medicine | | | 57% | Cardiovascular disease | 55% | Internal medicine | 57% |
|  | Cardiovascular disease | 52% | Cardiovascular disease | | | 54% | Internal medicine | 54% | Cardiovascular disease | 54% |
|  | Family medicine | 49% | Family medicine | | | 53% | Family medicine | 50% | Family medicine | 44% |
|  | Diagnostic radiology | 40% | Diagnostic radiology | | | 37% | Diagnostic radiology | 35% | Diagnostic radiology | 42% |
|  | Emergency medicine | 33% | Emergency medicine | | | 37% | Emergency medicine | 35% | Emergency medicine | 38% |

Abbreviations: CAD, coronary artery disease; cCTA, coronary computed tomography angiography; PET MPI, positron emission tomography myocardial perfusion imaging; SPECT MPI, single-photon emission computed tomography myocardial perfusion imaging.

#### Table S4: Proportion of patients receiving follow-up imaging by cohort

| **Cohort 1 (low pre-test risk, CAD diagnosis within 3 months of index test)** | **Stress echocardiography** | | **SPECT MPI** | | **PET MPI** | | **cCTA** | | **None** | |
| --- | --- | --- | --- | --- | --- | --- | --- | --- | --- | --- |
|  | Within 3 months | Within 1 year | Within 3 months | Within 1 year | Within 3 months | Within 1 year | Within 3 months | Within 1 year | Within 3 months | Within 1 year |
| **Stress echocardiography** | 2% | 3% | 4% | 6% | 0% | 0% | 4% | 4% | 97% | 93% |
| **SPECT MPI** | 0% | 1% | 2% | 4% | 0% | 0% | 2% | 2% | 97% | 95% |
| **PET MPI** | 0% | 0% | 0% | 1% | 1% | 3% | 1% | 1% | 98% | 95% |
| **cCTA** | 1% | 2% | 5% | 7% | 0% | 1% | 3% | 3% | 91% | 88% |
| **Cohort 2 (low pre-test risk, no CAD diagnosis following index test)** | **Stress echocardiography** | | **SPECT MPI** | | **PET MPI** | | **cCTA** | | **None** | |
|  | Within 3 months | Within 1 year | Within 3 months | Within 1 year | Within 3 months | Within 1 year | Within 3 months | Within 1 year | Within 3 months | Within 1 year |
| **Stress echocardiography** | 1% | 2% | 1% | 2% | 0% | 0% | 1% | 1% | 99% | 97% |
| **SPECT MPI** | 0% | 0% | 2% | 2% | 0% | 0% | 0% | 1% | 98% | 97% |
| **PET MPI** | 0% | 0% | 0% | 1% | 1% | 2% | 0% | 0% | 98% | 97% |
| **cCTA** | 1% | 1% | 1% | 2% | 0% | 0% | 1% | 2% | 97% | 95% |

| **Cohort 3 (high pre-test risk, CAD diagnosis within 3 months of index test)** | **Stress echocardiography** | | **SPECT MPI** | | **PET MPI** | | **cCTA** | | **None** | |
| --- | --- | --- | --- | --- | --- | --- | --- | --- | --- | --- |
|  | Within 3 months | Within 1 year | Within 3 months | Within 1 year | Within 3 months | Within 1 year | Within 3 months | Within 1 year | Within 3 months | Within 1 year |
| **Stress echocardiography** | 2% | 4% | 6% | 8% | 0% | 0% | 4% | 4% | 97% | 93% |
| **SPECT MPI** | 0% | 1% | 3% | 6% | 0% | 0% | 2% | 2% | 96% | 93% |
| **PET MPI** | 0% | 0% | 1% | 2% | 1% | 3% | 1% | 1% | 97% | 94% |
| **cCTA** | 1% | 2% | 6% | 9% | 0% | 1% | 3% | 3% | 90% | 86% |
| **Cohort 4 (high pre-test risk, no CAD diagnosis following index test)** | **Stress echocardiography** | | **SPECT MPI** | | **PET MPI** | | **cCTA** | | **None** | |
|  | Within 3 months | Within 1 year | Within 3 months | Within 1 year | Within 3 months | Within 1 year | Within 3 months | Within 1 year | Within 3 months | Within 1 year |
| **Stress echocardiography** | 1% | 2% | 2% | 3% | 0% | 0% | 0% | 0% | 99% | 97% |
| **SPECT MPI** | 0% | 0% | 2% | 3% | 0% | 0% | 0% | 0% | 98% | 96% |
| **PET MPI** | 0% | 0% | 0% | 1% | 1% | 2% | 0% | 0% | 98% | 97% |
| **cCTA** | 1% | 1% | 2% | 3% | 0% | 0% | 2% | 2% | 96% | 94% |

| **Cohort 5 (existing CAD diagnosis, no prior cardiac events)** | **Stress echocardiography** | | **SPECT MPI** | | **PET MPI** | | **cCTA** | | **None** | |
| --- | --- | --- | --- | --- | --- | --- | --- | --- | --- | --- |
|  | Within 3 months | Within 1 year | Within 3 months | Within 1 year | Within 3 months | Within 1 year | Within 3 months | Within 1 year | Within 3 months | Within 1 year |
| **Stress echocardiography** | 1% | 3% | 3% | 5% | 0% | 0% | 1% | 1% | 99% | 94% |
| **SPECT MPI** | 0% | 0% | 2% | 5% | 0% | 0% | 0% | 1% | 98% | 94% |
| **PET MPI** | 0% | 0% | 0% | 2% | 1% | 3% | 0% | 1% | 99% | 94% |
| **cCTA** | 1% | 1% | 4% | 7% | 0% | 1% | 2% | 3% | 93% | 89% |
| **Cohort 6 (existing CAD diagnosis, prior cardiac event within 1 year)** | **Stress echocardiography** | | **SPECT MPI** | | **PET MPI** | | **cCTA** | | **None** | |
|  | Within 3 months | Within 1 year | Within 3 months | Within 1 year | Within 3 months | Within 1 year | Within 3 months | Within 1 year | Within 3 months | Within 1 year |
| **Stress echocardiography** | 2% | 6% | 4% | 9% | 0% | 0% | 1% | 1% | 98% | 88% |
| **SPECT MPI** | 0% | 1% | 3% | 9% | 0% | 0% | 0% | 0% | 97% | 90% |
| **PET MPI** | 0% | 1% | 1% | 4% | 1% | 5% | 0% | 1% | 97% | 90% |
| **cCTA** | 1% | 2% | 4% | 8% | 0% | 1% | 2% | 3% | 93% | 87% |

| **Cohort 7 (existing CAD diagnosis, prior cardiac event within 1–2 years)** | **Stress echocardiography** | | **SPECT MPI** | | **PET MPI** | | **cCTA** | | **None** | |
| --- | --- | --- | --- | --- | --- | --- | --- | --- | --- | --- |
|  | Within 3 months | Within 1 year | Within 3 months | Within 1 year | Within 3 months | Within 1 year | Within 3 months | Within 1 year | Within 3 months | Within 1 year |
| **Stress echocardiography** | 2% | 5% | 4% | 7% | 0% | 0% | 0% | 1% | 99% | 92% |
| **SPECT MPI** | 0% | 1% | 3% | 7% | 0% | 0% | 0% | 0% | 97% | 92% |
| **PET MPI** | 0% | 0% | 1% | 2% | 1% | 5% | 0% | 0% | 98% | 93% |
| **cCTA** | 2% | 2% | 5% | 9% | 0% | 0% | 3% | 5% | 90% | 85% |
| **Cohort 8 (low pre-test risk, subsequent CAD diagnosis more than 3 months after index test)** | **Stress echocardiography** | | **SPECT MPI** | | **PET MPI** | | **cCTA** | | **None** | |
|  | Within 3 months | Within 1 year | Within 3 months | Within 1 year | Within 3 months | Within 1 year | Within 3 months | Within 1 year | Within 3 months | Within 1 year |
| **Stress echocardiography** | 1% | 3% | 2% | 6% | 0% | 0% | 1% | 3% | 99% | 90% |
| **SPECT MPI** | 0% | 1% | 2% | 5% | 0% | 0% | 0% | 2% | 98% | 93% |
| **PET MPI** | 0% | 0% | 0% | 2% | 1% | 5% | 0% | 1% | 98% | 92% |
| **cCTA** | 1% | 2% | 2% | 5% | 0% | 1% | 2% | 3% | 95% | 90% |

| **Cohort 9 (high pre-test risk, subsequent CAD diagnosis more than 3 months after index test)** | **Stress echocardiography** | | **SPECT MPI** | | **PET MPI** | | **cCTA** | | **None** | |
| --- | --- | --- | --- | --- | --- | --- | --- | --- | --- | --- |
|  | Within 3 months | Within 1 year | Within 3 months | Within 1 year | Within 3 months | Within 1 year | Within 3 months | Within 1 year | Within 3 months | Within 1 year |
| **Stress echocardiography** | 1% | 4% | 3% | 8% | 0% | 0% | 1% | 2% | 98% | 90% |
| **SPECT MPI** | 0% | 1% | 2% | 7% | 0% | 0% | 0% | 1% | 97% | 92% |
| **PET MPI** | 0% | 1% | 0% | 2% | 1% | 3% | 0% | 1% | 98% | 92% |
| **cCTA** | 0% | 2% | 4% | 8% | 0% | 0% | 1% | 3% | 94% | 87% |

Abbreviations: CAD, coronary artery disease; cCTA, coronary computed tomography angiography; PET MPI, positron emission tomography myocardial perfusion imaging; SPECT MPI, single-photon emission computed tomography myocardial perfusion imaging.

#### Table S5: Proportion of patients receiving downstream coronary angiography, by index test

| **Cohort** | **Stress echocardiography** | **SPECT MPI** | **PET MPI** | **cCTA** |
| --- | --- | --- | --- | --- |
| **Cohort 1 (low pre-test risk, CAD diagnosis within 3 months of index test) (N=110k)** | 31.0% | 31.3% | 15.4% | 17.4% |
| **Cohort 2 (low pre-test risk, no CAD diagnosis following index test) (N=622k)** | 1.3% | 2.9% | 2.2% | 1.1% |
| **Cohort 3 (high pre-test risk, CAD diagnosis within 3 months of index test) (N=141k)** | 36.5% | 38.2% | 24.2% | 21.5% |
| **Cohort 4 (high pre-test risk, no CAD diagnosis following index test) (N=571)** | 1.6% | 3.5% | 2.9% | 1.6% |
| **Cohort 5 (existing CAD diagnosis, no prior cardiac events) (N=818k)** | 9.9% | 15.0% | 14.9% | 15.9% |
| **Cohort 6 (existing CAD diagnosis, prior cardiac event within 1 year) (N=108k)** | 18.2% | 23.8% | 27.1% | 22.1% |
| **Cohort 7 (existing CAD diagnosis, prior cardiac event within 1–2 years) (N=50k)** | 5.4% | 8.0% | 8.8% | 8.5% |
| **Cohort 8 (low pre-test risk, subsequent CAD diagnosis more than 3 months after index test) (N=54k)** | 10.8% | 13.1% | 9.0% | 6.6% |
| **Cohort 9 (high pre-test risk, subsequent CAD diagnosis more than 3 months after index test) (N=83k)** | 11.9% | 14.1% | 11.5% | 8.6% |

Abbreviations: CAD, coronary artery disease; cCTA, coronary computed tomography angiography; PET MPI, positron emission tomography myocardial perfusion imaging; SPECT MPI, single-photon emission computed tomography myocardial perfusion imaging.
